# Rigorous Calibration of UAV-Based LiDAR Systems with Refinement of the Boresight Angles Using a Point-to-Plane Approach

**DOI:** 10.3390/s19235224

**Published:** 2019-11-28

**Authors:** Elizeu Martins de Oliveira Junior, Daniel Rodrigues dos Santos

**Affiliations:** Department of Geomatics, Federal University of Paraná, Curitiba, Paraná, 19001, Brazil; elizeuoliveirajunior@gmail.com

**Keywords:** UAV-based LiDAR systems, calibration parameters, boresight angles, point-to-plane approach

## Abstract

Advances in micro-electro-mechanical navigation systems and lightweight LIDAR (light detection and ranging) sensors onboard unmanned aerial vehicles (UAVs) provide the feasibility of deriving point clouds with very high and homogeneous point density. However, the deformations caused by numerous sources of errors should be carefully treated. This work presents a rigorous calibration of UAV-based LiDAR systems with refinement of the boresight angles using a point-to-plane approach. Our method is divided into a calibration and a parameter mounting refinement part. It starts with the estimation of the calibration parameters and then refines the boresight angles. The novel contribution of the paper is two-fold. First, we estimate the calibration parameters conditioning the centroid of a plane segmented to lie on its corresponding segmented plane without an additional surveying campaign. Second, we refine the boresight angles using a new point-to-plane model. The proposed method is evaluated by analyzing the accuracy assessment of the adjusted point cloud to point/planar features before and after the proposed method. Compared with the state-of-the-art method, our proposed method achieves better positional accuracy.

## 1. Introduction

Nowadays, UAVs (unmanned aerial vehicles) based on LiDAR (light detection and ranging) systems are one of the most cost-effective tools for a broad class of different applications such as digital building model generation [1], mapping [2], disaster management [3,4], forestry inventory [5,6,7], archaeological studies [8,9], power line inspection [10], and others. Due to their flexibility and mobility, they also have great potential for mapping and change detection. UAV-based LiDAR systems can rapidly capture georeferenced point clouds with very high point density. To that end, they combine the measurements of the laser unit (range and scanning mirror angles) and mounting calibration parameters (lever-arm and boresight misalignment) with the estimated platform trajectory obtained, as a function of time, from a GNSS (global navigation satellite system) and an INS (inertial navigation system) integrated into some sort of Kalman filter. Moreover, uncompensated effects in system calibration [11], systematic errors introduced from the key components of the integrated GNSS/INSS navigation system and the scanning mechanism [12], strong vibrations raised by its highly variable flight dynamics, and the restricted satellite visibility at very low flying heights [13] are limiting factors in the derivation of point clouds with high positional accuracy. In practice, there is no easy way to model such effects and to remove them from observations without calibrating the role of the system. Thus, a calibration procedure (or a strip adjustment) is required, which is the focus of this study.

Typically, existing approaches can be grouped into two different categories: (1) non-rigorous methods and (2) rigorous methods. Non-rigorous methods use the modeling of systematic errors in the object domain. They aim to estimate translational and rotational parameters between overlapping LiDAR strips by considering reference data information (if available) using point-based [14,15,16,17,18,19,20,21] or feature-based approaches [22,23,24,25]. In general, the identification of point features is difficult due to the irregular nature and limited spatial resolution of the LiDAR data [25]. An alternative point-based solution to solve non-rigorous strip adjustment tasks is done using a standard pairwise ICP (iterative closest point) algorithm [26,27]. First, the corresponding points are extracted directly from the overlapping area of the strips. Then, a rotation matrix R and a translation vector t (transformation parameters) are estimated by minimizing, iteratively, the discrepancies among corresponding features from overlapping LiDAR strips. The method is computationally non-attractive, and manual interventions might be necessary. A plane-based approach to assessing the discrepancies between overlapping LiDAR strips was proposed by Latypov [22]. The author derived the transformation parameters by comparing corresponding surfaces in the overlapping strips. Vosselman [13] introduced linear features such as gable roofs for the estimation of the transformation parameters between corresponding patches of overlapping LiDAR strips. In [25], the point-to-plane distances were used as observables in a linear model to estimate the transformation parameters and quality measures between points in one strip and their corresponding planes in the other. Typically, non-rigorous methods are done without the GNSS/INS trajectory data, offering a fast and automated solution. Moreover, in this way, some accuracy may be lost once the correction of laser points focuses on the effect of the systematic errors but not on their causes [28]. On the other hand, rigorous methods rely on the approach of sensor calibration parameters. In other words, the method does infer the causes of the detected systematic errors. The point cloud is rigorously modeled by considering the calibration parameters and the trajectory [11,13,29,30,31,32,33,34,35]. The method proposed by [11] estimates the calibration parameters conditioning the object coordinates of a group of points to lie on a common plane. The planar surfaces are manually extracted, and the calibration parameters are estimated together with the utilized planes. Moreover, this constraint constitutes a limiting factor in error propagation in the least-squares method. The time-dependent correction of trajectory errors is circumvented in [13], where the errors are modeled using a spline trajectory correction model. The iterative estimation task proposed by [13] is partly based on [27], where a point-to-plane distance is minimized through correspondences defined by the two closest points and their normal vectors. Therefore, additional ground-truth data collection is required to avoid absolute deformation of the ALS (airborne laser system) block. In [29], a two-step procedure for LiDAR system calibration was proposed. In this method, first, the discrepancies between overlapping LiDAR strips are obtained using only parallel strips. Thus, the impact of the present systematic errors on the LiDAR data is modeled. Second, the time-tagged LiDAR point cloud and the trajectory position data are used in a single-step procedure to estimate the system mounting parameters while the discrepancies between correspondence surfaces in parallel and non-parallel strips are reduced. The similarity measure is done using a point-to-patch corresponding model. The point-to-patch correspondences are obtained using a variant of the ICP algorithm, called ICPatch. Kilian et al. [31] collected tie and control points in overlapping areas of the single strips to calibrate the discrepancy over strips. The mathematical model used was a linear drift equation for positions and attitudes. The result of this procedure is a set of 12 unknown transformation parameters for every single strip. The correct boresight angle error is obtained using the rotation matrix, and the translation vector is computed for each point. In the rigorous calibration of the LiDAR system proposed by [32], the mounting parameters, the bias in the measured ranges, and the scale factor in the mirror angle are estimated using identified discrepancies between conjugate surfaces. To deal with the irregular nature of the LiDAR surfaces, the implemented method in [32] uses a point-to-patch approach as devised by [29]. In the study by Ravi et al. [33], a geometric calibration method with stationary Velodyne VLP-16 Puck HI-RES was performed using conjugate planar/linear features obtained from different flight lines. The rigorous method proposed in [33] utilizes designed calibration boards covered by highly reflective surfaces to facilitate automated identification of feature correspondences, and it has restrictions in terms of flight configurations. Li et al. [24] used points scanned from different directions and strips to carry out boresight self-calibration in mobile LiDAR scanning systems and UAV-based LiDAR scanning systems. The registration of overlapping strips was performed using a variant of the ICP algorithm proposed by [35]. Zhang et al. [36] proposed an approach using an automated boresight angular error rectification method for the UAV LiDAR system based on laser intensity information. In this method, first, the SIFT (scale feature invariant transform) algorithm is applied to extract the key points from the intensity image and obtain the matched points from the overlapping intensity images. Afterwards, the matched points in 3D are determined using a 2D-to-3D mapping strategy. Thus, an approximate solution to the boresight angles is obtained using the LSM (least square mean). Then, the boresight angular errors are iteratively updated. 

This study focuses on the rigorous calibration of UAV-based LiDAR systems, specifically investigating a point-to-plane strategy that is capable of estimating the calibration parameters and refining the boresight angles to obtain an accurate 3D point cloud. The contribution of our proposed method is two-fold. First, we propose a constraint that conditions the centroid of a plane segmented to lie on its corresponding segmented plane to estimate the calibration parameters. This guarantees that correct calibration parameters are estimated even with the high positioning noise level of the GNSS/INS trajectory. As described by Skaloud and Lichti [11], the recovery of plane parameters together with the calibration parameters does not require any additional surveying campaign for system calibration. Second, we develop a new corresponding point-to-plane model to minimize the point/plane distance between tie feature correspondences from different LiDAR strips. The proposed model is based on the extraction of naturally existing corresponding points. Three-dimensional points are generated via point intersection between the orthogonal projection from a point in the reference plane with its corresponding target plane. The results are analyzed by quantifying the normal distance of point-to-plane features before and after the proposed solution. Section 2 describes the materials and proposed method for rigorous calibration of UAV-based LiDAR systems. Section 4 focuses on the experimental results and analysis. Finally, the conclusions and recommendations for future work are provided in Section 5. 

## 2. Data and Methods

### 2.1. Study Area and Data Acquisition

**Figure 1:**
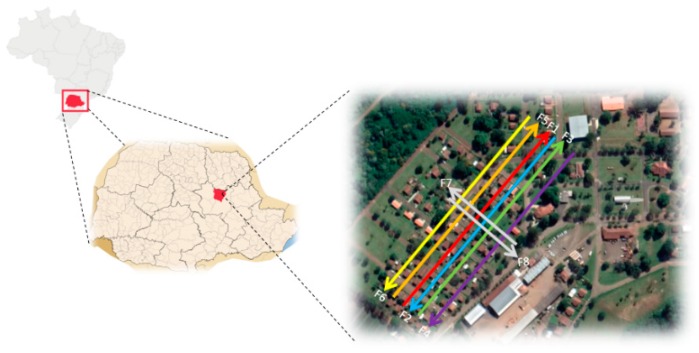
The geographic location of the study area and the unmanned aerial vehicle (UAV) flight strips acquired in the experiment; each color line indicates a flight line (F1-F8).

The study area is located in a farming area in the southwest of Paraná state, Brazil (–25°45′16”S, –49°23′39” E). The UAV-based LiDAR payload contains a Velodyne VLP-16 Puck HI-RES, which has 16 laser beams that are aligned over the range of +10° to –10°. It provides total vertical and horizontal fields of view of 20° and 30°, respectively. It can scan up 300.000 points per second with a range of 100 m with an accuracy better than 0.03 m [37]. The laser scanner is mounted on a DJI S1000 UAV platform integrated with an Applanix APX-15 UAV, whose accuracy attained after post-processing with the POSPac software from Applanix is 0.025° for pitch/roll and 0.08° for yaw, and the position accuracy is 0.02–0.05 m [38]. To derive point clouds with high positional accuracy, we must estimate the mounting parameters of the LiDAR unit concerning the onboard GNSS/INS. Thus, we collected data from eight overlapping flight line directions from North-to-South and two opposite flight line directions (Figure 1). 

**Figure 2:**
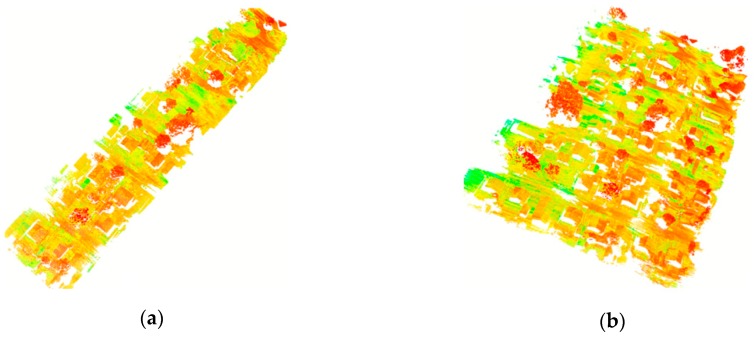
Examples of point clouds derived from the proposed method for one strip (**a**) and three adjusted LiDAR strips (**b**).

The UAV flew at a low altitude of 30 m above ground level at a cruising speed of 4.5 m/s. The laser scanner was set with a scanning rate at 400 kHz, generating ~300 pts/m^2^ for each LiDAR strip (Figure 2). Each flight trajectory obtained with the integrated LiDAR onboard GNSS/INS unit contained x, y, and z laser coordinates, the laser intensity, range, vertical angle, horizontal angle, latitude, longitude, elevation, roll angle, pitch angle, and heading angle. Table 1 shows the percentages of overlap between the LiDAR strips and their flight line directions.

### 2.2. Method

**Figure 3:**
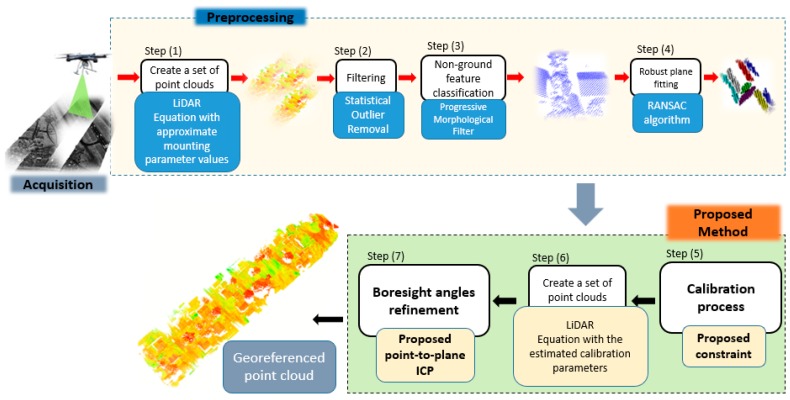
The framework of the proposed rigorous calibration of UAV-based LiDAR systems.

Figure 3 shows the generic structure of our proposed framework for rigorous calibration of UAV-based LiDAR systems. In particular, it uses a point-to-plane error metric that requires several considerations concerning the optimal choice of parameters, as presented by Skaloud and Litch [11]. However, to deal with UAV data requirements, we propose a new minimum distance criteria constraint, which mostly restricts the centroid of a plane to its corresponding plane. We also propose the use of a point-to-plane approach to refine the estimated boresight angles. 

In Figure 3, our framework is divided into seven steps. First, given a set of flight trajectories derived from our UAV-based LiDAR system, we created a set of LiDAR strips with the flight trajectory data and the initial values of the mounting parameters. Our framework used the statistical outlier removal method [39] to detect and remove outliers. Then, for each pair of LiDAR strips, the objects were classified on the ground and non-ground points using a progressive morphological filter [40]. Herein, both the ground and vegetation points were removed from the LiDAR strips while buildings were preserved. Thus, a robust gabled roof plane fitting was realized via the RANSAC (random sample consensus) algorithm [41]. Then, the region growing algorithm developed by [42] was applied to plane detection. The plane parameters were estimated based on principal component analysis (PCA). The subsequent task consisted of estimating the calibration parameters (boresight angles and range offset) using a proposed point-to-plane approach with the minimum distance criteria constraint. Finally, a corresponding point-to-plane model was proposed for the refinement of the boresight angles, allowing a georeferenced point cloud with high positional accuracy to be obtained. 

#### 2.2.1. Point Cloud Generation Using the LiDAR Equation

Typically, a UAV-based LiDAR system involves three coordinate systems: the mapping frame (*m-frame*), INS body frame (*b-frame*), and laser unit frame (*l-unit*). A *j-th* point in the m-frame ($p_{j}^{m}$) was constructed in the mapping coordinate system using equation (1) using the trajectory data derived from the UAV-based LiDAR system (GNSS/INS unit position and orientation) and the initial values of the mounting parameters, as follows:

(1) $$p_{j}^{m}=g_{nav}^{m}\left(t\right)+R_{b}^{m}\left(t\right)\left[{Ω}_{l}^{b}r_{j}^{l}+g_{nav}^{b}\right]$$

where $g_{nav}^{m}\left(t\right)$ represents the coordinate vector at time t of the GNSS/INS in the m-frame, $R_{b}^{m}\left(t\right)$ is the rotation matrix between the body frame (b-frame) and the m-frame, ${Ω}_{l}^{b}$ is the skew-symmetric part of the boresight matrix between the laser unit (l-unit) and the b-frame, $r_{j}^{l}$ denotes the coordinate vector of *j-th* point in the l-unit, and $g_{nav}^{b}$ is the lever arm vector. 

For the laser unit frame, the origin was defined at the laser beam firing point, and the z-axis was along the axis of rotation of the laser unit. Therefore, for the spinning multi-beam laser unit considered, each *j-th* point in the *l-unit* was obtained by

(2) $$r_{j}^{l}=\;\left(\begin{array}{c} \rho \left(t\right)\;cos\;\beta \left(t\right)cos\;\alpha \left(t\right)\\ \rho \left(t\right)\;cos\;\beta \left(t\right)sin\;\alpha \left(t\right)\\ \rho \left(t\right)\;sin\;\beta \left(t\right) \end{array}\right)$$

where each laser beam was fired at a fixed vertical angle (β), the horizontal angle (α) was determined based on the rotation of the laser unit, and the range (ρ) was defined by the distance between the firing point and its footprint. Note that, to derive point clouds with high positional accuracy, the estimation of the calibration parameters is a necessary step. 

#### 2.2.2. Point Cloud Processing

Due to the varying data density presented in LiDAR data, there are measurement errors that can lead to sparse outliers. This can skew and mislead the future process, corrupting the registration results even more. In this study, the statistical outlier removal technique proposed by [39] was used. The outliers were detected by analyzing a query point $p_{q}=\left\{x,y,z\right\}$ with respect to its surrounding neighbors, k. Given a reference strip (ℵ), the mean distance $d_{p}$ between each $p_{q}\in ℵ$ and its k neighbors were computed. Then, a filtered strip (${ℵ}^{\prime }$) was obtained using the mean $\mu_{k}$, the standard deviation $\sigma_{k},$ and the density restrictiveness factor α of the distribution over $d_{p}$ space for the entire ℵ. To access the set of k neighbors of $p_{q}$, a radius r search had to be defined. 

Since our rigorous calibration method is based on a point-to-plane corresponding model, an algorithm to remove the ground objects and the vegetation from LiDAR strip needed to be used. To eliminate non-ground objects, we used a progressive morphological filter (PPF), as proposed by Zhang et al. [40]. Building objects were classified as non-ground points, and the RANSAC algorithm was used to remove the vegetation from LiDAR data. The general workflow of the PPF+RANSAC algorithm can be summarized as follows: (1) Open a window within a predefined size ($w_{1}$) around a point p; (2) find the maximum and minimum elevation values in $w_{1}$. The dilation represents the maximum value, and erosion denotes the minimum value in w; (3) apply erosion followed by a dilation process. The large non-ground objects remain while small vegetation is removed. For the ground objects, features smaller than $w_{1}$ are smoothed and replaced by the minimum elevation value, and an initial filtered surface can be obtained; (4) compute the height difference between the original LiDAR data and the initial filtered surface ($dh_{,fil}$). Estimate a height difference threshold ($dh_{T,fil}$) based on the terrain slope. If $dh_{,fil}>dh_{T,fil}$, then p is classified as a non-ground object; (5) the window size is linearly enlarged using $w_{k}=2kb+1$, where k=1,2,…N, and b is the initial window. Again, execute erosion followed by dilation. A new filtered surface is determined; (6) the ground objects can be removed by gradually increasing w and by computing new filtered surfaces until w is greater than the size of the largest object. To separate the gabled roofs from the vegetation, we executed the RANSAC algorithm because vegetation points can be detected as outliers in the planar surface extraction task. 

To segment gabled roofs in the overlapping strips, we used the 3D region growing algorithm proposed by Rabbani et al. [42]. For this, given a point p, its k-nearest neighbors were searched with the *K*-d trees strategy [43]. Then, the Lagrange multipliers were used to fit the surface planes [44], achieving eigenvalues representing an approximation of the normal vector $n=\left\{n_{x},n_{y},n_{z}\right\}$ of the unit length. The region growing procedure was based on the joint effort of local connectivity and surface smoothness. First, the points in a segment were locally connected using the k-d tree technique. Second, an angular threshold value $\theta_{T}$ between the normal vectors of the current point $n_{c}$ and its neighborhood points $n_{b}$ was used. For $∥n_{c}\cdot n_{b}∥<cos\left(\theta_{T}\right),$ the analyzed neighbor point was added to the segmented surface. A residual value $res_{T}$ was also used to make sure that smooth areas were broken on the edges [42]. Since reference and target planes were segmented, the points of the plane were used to estimate the surface normal and the distance from the origin to the plane (d) based on the first-order 3D plane fitting method. Thus, a robust gabled roof plane fitting was realized using the RANSAC algorithm.

#### 2.2.3. Estimation of the Calibration Parameters Using the Minimum Distance Criteria Constraint

Similar to Skaloud and Litch [11], we recovered the plane parameters together with the boresight angles and range offset in a combined adjustment model. In this case, no additional surveying campaign apart from the calibration flight itself needed to be performed for the system calibration [11]. As a prerequisite, the authors recommend that many planar features with different spatial orientations must be used to achieve good calibration results. Thus, they proposed a constraint conditioning the object coordinates of the group of points to lie on a common plane. In practice, the unit length of the direction cosines of the plane’s normal vector is fixed. Statistically speaking, the proposed constraint enforces the high reliability of the extracted plane. As a consequence, it constitutes a limiting factor in the error propagation into the least-squares method. Since UAV-based LiDAR systems produce noisy point clouds due to the strong vibrations raised by their highly variable flight dynamics and the restricted satellite visibility at very low flying heights, the constraint devised and implemented by [11] cannot guarantee consistency in the estimated solution. To overcome this limitation, we propose a constraint conditioning the centroid of a segmented plane in ${ℵ}^{\prime }$ (filtered-reference LiDAR strip) to lie on its corresponding segmented plane in Γ′ (filtered-search LiDAR strip). The functionality of the proposed constraint can act as a reliable estimate. 

For this work, given a set of object points *j* expressed by its coordinates $x_{j}$, $y_{j},\;z_{j}$ that lie on a plane π, the observation equation can be written as

(3) $$\langle \pi^{T},\;{\left[\begin{array}{cc} p_{j}^{m} & 1 \end{array}\right]}^{T}\rangle$$

where $\pi ={\left(n^{T},-d\right)}^{T}$ is the homogeneous representation of the plane defined by a normal vector $n={\left(n_{x},\;n_{y},n_{z}\right)}^{T}$ of unit length and its perpendicular distance d from the origin. 

By substituting the LiDAR Equation (1) into the observation Equation (3), we can obtain the following expression:

(4) $$\langle \pi^{T},\;g_{nav}^{m}\left(t\right)+R_{b}^{m}\left(t\right)\left[{Ω}_{l}^{b}r_{j}^{l}+g_{nav}^{b}\right]\rangle .$$

We present a constraint based on the minimum distance criterion forcing the centroids (c) to belong to the correspondent planes in other strips:

(5) $$\langle n,\;{\left[\begin{array}{cc} c_{j}^{m} & 1 \end{array}\right]}^{T}\rangle$$

where $c_{j}^{m}={\left(x_{j},\;y_{j},z_{j}\right)}^{T}$ is the centroid of the corresponding plane segmented in Γ′.

Due to the system mounting platform, the calibration parameters used were the boresight angles Δω, Δφ, Δκ besides the range offset of the different lasers available. The arrangements of these parameters were separated in different scenarios aiming to study the best choice for UAV platforms; therefore, all of these parameters were considered in the calibration process. Since the observations and parameters of the point-on-plane functional mathematical model are not separable and each constraint provides more than one observation, the Gauss–Helmert adjustment model needed to be used. In that case, we used the linearization process model presented by [11].

#### 2.2.4. Point-to-Plane Matching Procedure

**Figure 4:**
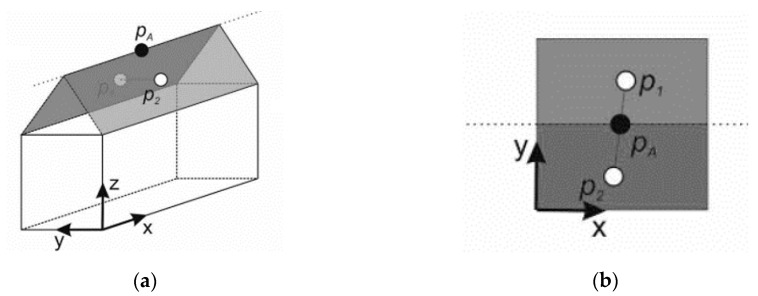
Illustration of the proposed point-to-plane matching procedure: (**a**) perspective view and (**b**) aerial view of the virtual points.

The proposed procedure for establishing the matching between corresponding point-to-plane pairs is explained in this section. As shown in Figure 4, a virtual point ($p_{A}$) can be determined from the intersection of the line segment defined by $p_{1}=\left[p_{1_{x}\;}\;p_{1_{y}}\right]$ (centroid of the left plane of the gabled roof) and $p_{2}=\left[p_{2_{x}\;}\;p_{2_{y}}\right]$ (centroid of the right plane) with the projection of the 3D straight line onto the horizontal plane. 

For the current work, the matching was performed in a direct manner. The correspondence between the virtual points ($p_{A}$) in ${ℵ}^{\prime }$ and the virtual points ($p_{A}\prime$) in Γ′ was established by determining the $p_{A}\prime$ with the shortest distance to the $p_{A}$ and the normal vector of the segmented plane from $p_{A}\prime$ with the shortest inclination to the normal vector of the segmented plane from $p_{A}$. To be considered a correct correspondence, the test results needed to be less than a given threshold. 

#### 2.2.5. Boresight Angle Refinement Using a Point-to-Plane Corresponding Model

**Figure 5:**
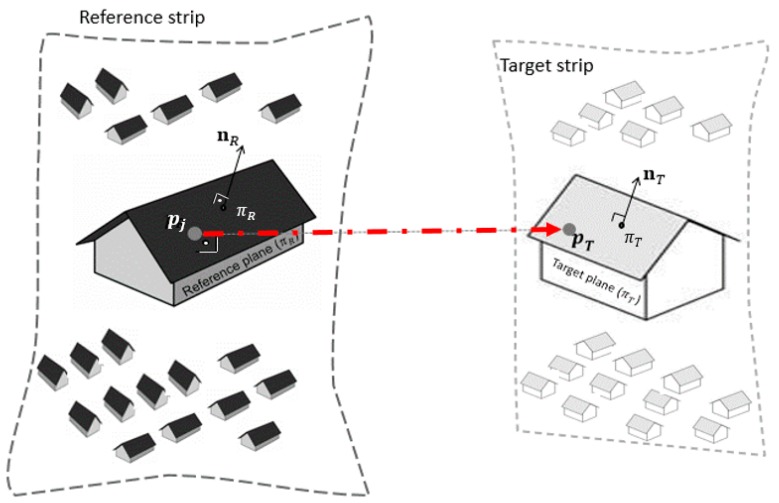
The perpendicular projection of a point in the reference plane to the corresponding target plane in the adjacent strip.

Once the correspondences had been established between strip pairs, the transformation parameters were estimated. Thus, we introduced a point-to-plane corresponding model with the aim of minimizing the sum of the distance between points and corresponding planes for alignment between strip pairs. Since each point ($p_{T}$) in the target plane ($\pi_{T}$) was computed via line-to-plane intersection (see Figure 5), our algorithm did not need to perform local approximations. 

As shown in Figure 5, for each point $p_{j}$ from ${ℵ}^{\prime }$, a point $p_{T}$ was computed in Γ′ via line-to-plane intersection. This 3D point intersection was formed by orthogonal projection (dashed line) from $p_{j}$ to the target plane $\pi_{T}$. Thus, a system of linear equations that could be used to solve the 3D coordinates of $p_{T}$ was developed as follows:

(6) $$\{\begin{array}{c} n_{T}p_{T}=d_{T}\;\\ p_{T}=p_{j}+sn_{T} \end{array}$$

where s denotes a scalar and $n_{T}={\left(n_{x},n_{y},n_{z}\right)}^{T}$ is the normal vector of the plane and its perpendicular distance $d_{T}$ from the origin.

In Equation (6), the first system equation is formulated using the corresponding target plane, while the second one represents the projected line from point $p_{j}$ in the reference plane passing through $p_{T}$ in the corresponding target plane. By substituting the first system equation into the second one, Equation (6) can be rewritten as follows:

(7) $$\{\begin{array}{c} s=d_{T}-n_{T}p_{j}\\ p_{T}=p_{j}+\left(d_{T}-n_{T}p_{j}\right)n_{T} \end{array}.$$

As $p_{T}$ is the orthogonal projection of $p_{j}$ in the corresponding plane, the distance between $p_{T}$ and $p_{j}$ represents the distance between $p_{j}$ and the target plane defined by its perpendicular distance $d_{T}$ from the origin and its normal vector $n_{T}$. In other words, Equation (7) represents a straight line from $p_{j}$ to $p_{T}$. Thus, to approximate the points of the planes, we have to minimize the following expression:

(8) $$e=\overset{n}{\underset{i=1}{\sum }}∥(d_{T}-n_{T}{}^{T}p_{j})n_{T}{∥}^{2}$$

where e is the point-to-plane error metric. 

By substituting Equation (1) in $p_{j}$ of the equation from (8) and rearranging the terms of this equation, we can obtain the following expression:

(9) $$e=\overset{n}{\underset{i=1}{\sum }}∥(d_{T}-n_{T}{}^{T}\left[g_{nav}^{m}\left(t\right)+R_{b}^{m}\left(t\right)\left[{Ω}_{l}^{b}r_{j}^{l}+g_{nav}^{b}\right]\right])n_{T}{∥}^{2}.$$

Equation (9) takes the form Ax+Bv+w=0, where A is the coefficient matrix containing the partial derivatives in (9), i.e., ${Ω}_{l}^{b}$(Δω, Δφ, Δκ), B is the coefficient matrix containing the partial derivatives of the same functional taken with respect to the observations, v is the residual vector, w is obtained by evaluating the condition equation with the observation and the approximate values of the unknowns, and x represents the corrections to the approximate values of the unknown parameters. The objective of the adjustment is to minimize the sum of all squared point-to-plane distances. Since at least four pairs of corresponding planes are obtained, the boresight angles are refined.

## 3. Results

### 3.1. Gabled Roof Extraction

The LiDAR strips’ data were captured with the Velodyne VLP-16 Puck HI-RES laser scanner integrated with an Applanix APX-15 onboard a DJI S1000 UAV platform. These data were de-noised with the statistical outlier removal algorithm to remove outliers. After that, the objects were classified on the ground and non-ground points using a progressive morphological filter (PPF), in which only the buildings were preserved. Next, a robust gabled roof plane fitting was realized with the RANSAC algorithm. After the plane extraction, a region growing algorithm was applied to segment the planes, and its parameters were obtained using PCA. Table 2 describes the parameters used for RANSAC, PPF, and the robust gabled roof plane fitting (RGF) method. 

The described tasks were performed using the proposed framework. The quality of the extracted planes was determined based on the tolerance used in the RANSAC algorithm, and the robust gabled roof plane method was applied for the planar feature extraction. Then, highly reliable gabled roof planes were used to estimate the calibration parameters. 

### 3.2. Benefits of the Proposed Rigorous Calibration Method

#### 3.2.1. Estimation of the Calibration Parameters

To obtain the 3D point clouds, we processed the trajectory data captured with our UAV-based LiDAR system using Equation (1). As a proof-of-concept of our calibration strategy, we made use of eight UAV-based LiDAR overlapping strips. The method was evaluated with three different scenarios (A, B, and C), where each one contained different calibration parameters to be estimated, as shown in Table 3. Note that, because the Velodyne VLP-16 Puck HI-RES LiDAR system contained a spinning multi-beam laser unit, each laser beam fired was set as a different parameter (Δρ).

To estimate the calibration parameters, the point-to-plane matching procedure was done semi-automatically. On the other hand, first, the correspondences between planes were established using our matching strategy (see Section 2.2.4). Afterwards, the false positives were manually eliminated. For scenario A, only the boresight angles were estimated. In scenario B, the boresight angles and the range offset were considered in the calibration procedure. For scenario C, the methodology proposed by Skaloud and Lichti [11] was used, where the boresight angles, the range offset, and the parameters of the planes are estimated simultaneously. The initial values of the boresight angles were set as a vector of zeros, and the lever arm was obtained with a topographic survey. The results for the calibration parameters for scenarios A, B, and C are listed in Table 4.

As can be observed in Table 4, the standard deviation of the boresight angles lies within the accuracy expected according to the hardware components. The resultant calibration error parameters were then substituted into Equation (1), and adjusted LiDAR strips were achieved. 

#### 3.2.2. Refinement of the Boresight Angles

**Figure 6:**
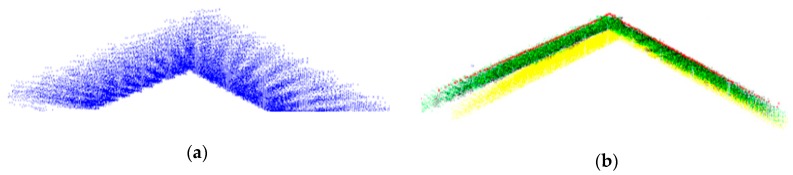
Visual check of the performance of the point cloud after calibration with the proposed method. (**a**) Target gable roof profile before the rigorous calibration method. (**b**) Target gable roof profile after the proposed method using scenario A (yellow points), scenario B (black points) and scenario C (green points), with the red line being the ground reference obtained with a Leica ALS60 airborne LiDAR sensor.

The framework used for gable roof extraction was performed for the adjusted LiDAR strips, and our point-to-plane matching procedure was done automatically. Equation (9) with the least-squares mean was applied to refine the boresight angles, and accurate 3D point clouds were achieved after the refinement task. A visual check of a gable roof profile is shown in Figure 6, which indicates a major improvement using the calibration parameters obtained with scenarios B and C. 

In Figure 6, although scenarios B and C present similar levels of performance, in scenario C, the calibration parameters were estimated jointly with the parameters describing the involved planes, as presented in [11]. In this case, the number of unknowns changed with the number of point-to-plane correspondences used in the calibration procedure [29]. This was in contrast to scenario B, where only the calibration parameters were estimated. Thus, when compared with scenario C, the unknown parameters did not depend on the number of point-to-plane correspondences used in the calibration procedure. 

#### 3.2.3. Accuracy Assessment

**Figure 7:**
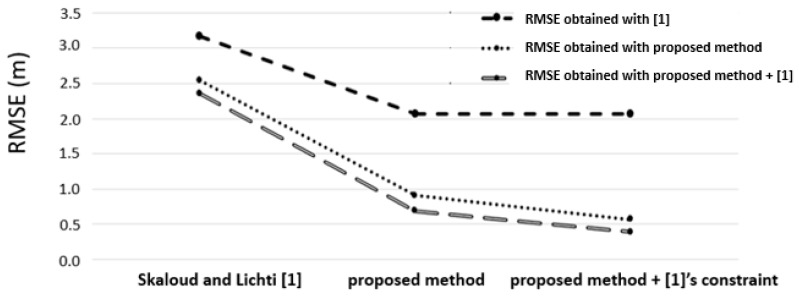
RMSE before and after the calibration. The x-axis indicates the methods applied, and the y-axis denotes the RMSE of the point-to-plane distances.

The overall goal of our framework was to estimate the calibration system parameters and refine the boresight angles. We thus started from a raw trajectory flight and assessed the quality of the final point cloud. Target gable roofs obtained with the refined boresight angles were directly compared to the ground reference gable roofs. The ground reference used was obtained with a Leica ALS60 airborne LiDAR sensor, model CUS6 INS, with the following absolute accuracies positioned between 5 and 30 cm: roll and pitch < 0.0025°, heading < 0.005°. The point cloud had an approximate density of 5 points/m^2^. As a sanity check, we compared our rigorous calibration method with the method of Skaloud and Lichti [11]. Given a set of segmented planes in the ground reference and the final point cloud, we first calculated the RMSE (root mean square error) of the normal distance of points from their corresponding planes before and after the rigorous calibration procedure obtained with both our method and the method of [11]. Figure 7 shows the RMSE values of our proposed framework and the RMSE values obtained with [11]. We still achieved suitable RMSE values by combining our proposed constraint with the method of Skaloud and Lichti [11]. Second, we calculated histograms of the point-to-plane distances before and after the procedure for results obtained with our method and with that of [11]. 

**Figure 8:**
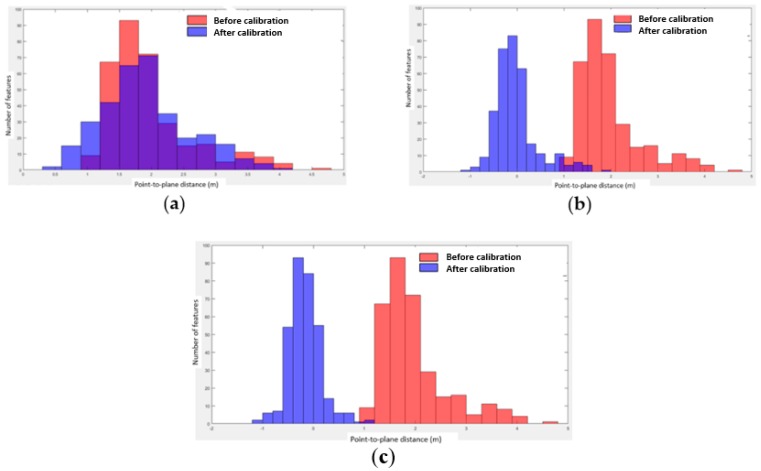
Histograms of the point-to-plane distances before and after the calibration procedure: (**a**) result obtained with [11], (**b**) result obtained with scenario B and (**c**) result obtained with scenario C.

As can be observed in Figure 7, the proposed method enabled a more refined point cloud than the approach proposed by [11], leading to more accurate data. As mentioned previously, our calibration strategy was based on conditioning the centroid of a segmented plane to lie on its corresponding segmented plane to provide a reliable estimate. We also incorporated the geometric constraint proposed in [11] into the proposed method, and the RMSE was slightly improved. Figure 8 shows the histograms of the point-to-plane distances before and after the rigorous calibration procedure.

It can be seen in Figure 8a, using the method outlined with Skaloud and Lichti [11], the RMSE between the point-to-plane distances was reduced to less than 12% of the value before the calibration procedure. The behavior in [11] was trajectory data-dependent, and in UAV-based LiDAR data, noised point clouds were produced due to the strong vibrations raised by its highly variable flight dynamics and the restricted satellite visibility at very low flying heights. In contrast, our proposed method achieved an RMSE higher than 90% for scenarios B and C (see Figure 8b,c, respectively). Closer inspection revealed that most of the improvement came from incorporating the proposed constraint, which indicates a reliable estimate for noised UAV-based LiDAR data. The point-to-plane approach with a large number of reliable corresponding features in the least-squares estimation model (9) also provided a significant refinement of boresight angles. In addition, the histograms in Figure 8b,c show an almost normal distribution, which means that the systematic errors were successfully eliminated. 

## 4. Discussion

As mentioned in [25], the identification of point features is difficult due to the irregular nature and the limited spatial resolution of the LiDAR data. Thus, we used planar surfaces identified in overlapping strips as primitives for the corresponding model. Although this requires preprocessing of the LiDAR point cloud, such as segmentation and plane fitting procedures, the planarity is not central to our method, as pointed out in [11]. In particular, by conditioning the centroid of a plane segmented to lie on its corresponding segmented plane, we can guarantee that the calibration parameters are correctly estimated even when the GNSS/INS trajectory is highly noised. This means that our field calibration can be successfully arranged in urban areas and is also suitable for rural areas (i.e., planar features can be found in natural terrains such as soccer fields). 

**Figure 9:**
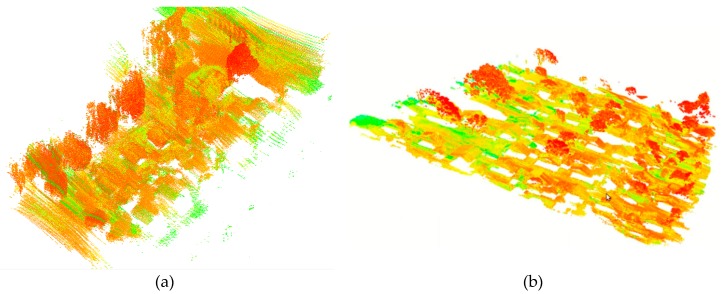
3D point clouds derived before the boresight angle calibration (**a**), and after the boresight angle refinement (**b**).

The primary goal of the rigorous calibration is to weed out systematic errors in the system parameters. Although not investigated here, our method is able to simultaneously estimate the lever-arm offset and the boresight angles, since optimal flight configuration design must be available, as described in [29]. To analyze the effect of the GNSS/INS trajectory in the point cloud, Figure 9 shows the derived point cloud before the boresight angle calibration (Figure 9a) and after the boresight angle refinement using the proposed method. 

**Figure 10:**
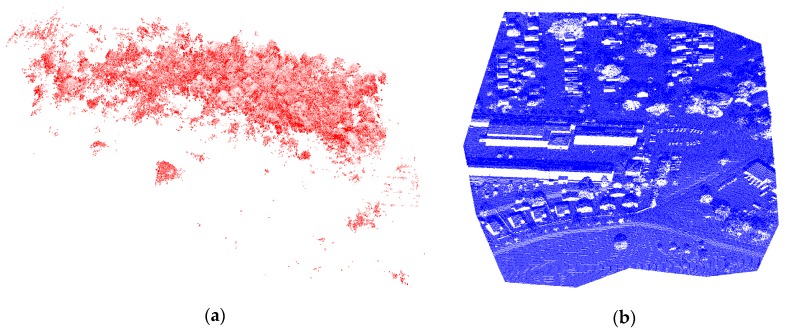
3D point clouds derived from the proposed method for applications in forestry inventory (**a**) and mapping or 3D modeling (**b**).

A visual inspection of point clouds generated before boresight angle calibration (see Figure 9a) revealed that a noised point cloud is derived from the GNSS/INS trajectory. Noise only affects the proposed method during plane extraction. To minimize the possibility of outliers, the statistical outlier removal technique [39] was used. It was also useful to eliminate false objects formed by highly reflective objects. Through visual inspection of the point cloud shown in Figure 9b, the proposed method can remove most of the boresight errors caused by uncompensated effects in system calibration. Some misalignments between different scanning strips can be observed; this is likely a result of the amount of uncertainty from LiDAR and GNSS/INS units used here. The results can be improved with higher sensor units providing a higher measurement accuracy. The presented approach is the first to adopt a closed-form solution. This method can generate 3D point clouds for one of the domains in which its use is expanding, i.e., the forestry and mapping sectors, as depicted in Figure 10. 

Figure 10 shows the derived point clouds with high positional accuracy obtained with our proposed method.

### 4.1. The Influence of the Proposed Constraint

The constraint conditioning the centroid of a plane segmented to lie on its corresponding segmented plane without an additional surveying campaign has a great impact on the estimation of calibration parameters. To explore the influence of this constraint, we compared our method with the method of [11] by analyzing the percentage of average errors obtained after each estimate solution. The statistical results are shown in Table 5. 

It can be seen that it is not necessary to estimate the parameters of the plane together with the calibration parameters when the proposed constraint is used. An important observation in this context is that the parameters associated with the involved planes are not part of the unknown variables. Typically, this method is less time-consuming in terms of the normal equation matrix inversion.

As a general comment, we point out that the proposed constraint in the calibration procedure delivers explicit information about platform stability. That is, for each pair of point-to-plane correspondences, we included a condition to provide a reliable estimate. In a practical sense, our method can attenuate the angular boresight errors caused by strong vibrations raised by highly variable flight dynamics from UAV-based LiDAR. 

### 4.2. The Influence of the Proposed Refinement Approach

**Figure 11:**
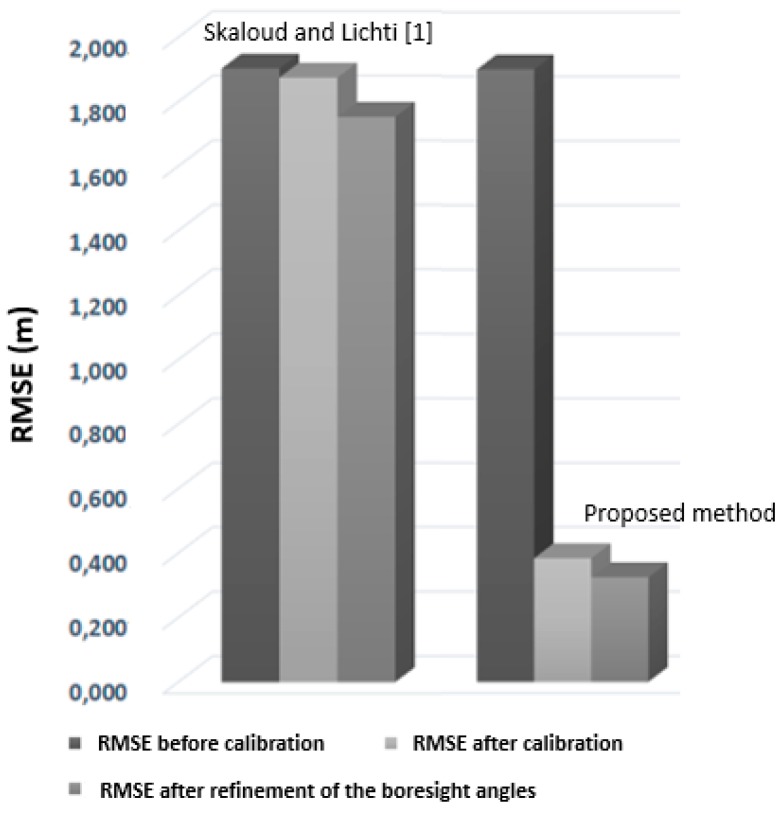
Absolute vertical accuracy obtained with the calibration parameters proposed by [11] and with the proposed method.

The calibration of UAV-based LiDAR systems is not a goal in itself but is only a precursor for the refinement of the boresight angles. Thus, we refined the boresight angles using the proposed point-to-plane corresponding model presented in Equation (9). To assess the absolute vertical accuracy of the final point cloud obtained with the proposed method, we calculated the difference between the virtual points $p_{A}$ Additionally, their corresponding points were collected in the reference ground. The results of the vertical offsets before and after each task of our proposed method are depicted in Figure 11. 

As illustrated in Figure 11, the vertical offset was better than 40 cm after the calibration task, while a vertical offset better than 30 cm was achieved after the proposed refinement task. The experimental results show that the vertical offset after boresight refinement was remarkably reduced compared with that of [11]. 

## 5. Conclusions

This paper presents a rigorous calibration method for UAV-based LiDAR systems involving the refinement of boresight angles using a point-to-plane approach. Conditioning the centroid of a plane to lie on its corresponding segmented plane corrected 80% of the systematic errors in our test area, on average. The calibration parameters do not need to be estimated together with the plane parameters; still, about 20% of these errors could not be reduced. This problem comes from the strong vibrations of UAV platform, the low quality of the integrated GNSS/INS navigation system, and the scanning mechanism. However, it can be improved by refining the boresight angles with our point-to-plane approach.

When the geometric constraint proposed by [11] was included, the RMSE was slightly improved. However, it cannot be used alone for UAV-based LiDAR data. A few problems should be addressed further. The segmentation process is time-consuming because it is iterative. Further research could introduce new strategies for plane fitting and matching procedures. In addition, a more stable UAV platform could be implemented, which could immensely reduce the systematic errors. 

## Figures and Tables

**Table 1:** Overlapping LiDAR (light detection and ranging) strips.

Pair of Strips	Overlap (%)	Flight Line Direction
F1-F2	100	North–South
F2-F3	70	North–South
F3-F4	50	North–South
F2-F4	20	North–North
F1-F5	70	North–North
F5-F6	50	North–South
F1-F6	20	North–South
F7-F8	100	West–East

**Table 2:** Input parameters used to segment planar features.

	**PPF**	
Cell size = 1 m; base of the exponential window = 2 cells; increment step for windows = 2 m; slope = 5%; maximum threshold = 10 m		
	**RANSAC**	
Minimum number of inliers = 15; tolerance = 10 cm; number of consensus = 10; angle minimum of the plane = 10 degrees, maximum angle of the plane = 80 degrees		
	**RGF**	
Minimum size = 40; maximum size = 500; angular threshold = 1 degree; outlier removal ratio = 4 m		

**Table 3:** Calibration parameters.

	**Scenario A**	
{ Δω, Δφ, Δκ }		
	**Scenario B**	
$\left\{\;\Delta \omega ,\;\Delta \varphi ,\;\Delta \kappa ,\;\Delta \rho_{1},\;\Delta \rho_{2},\ldots ,\;\Delta \rho_{16}\right\}$		
	**Scenario C**	
$\left\{\;\Delta \omega ,\;\Delta \varphi ,\;\Delta \kappa ,\;\Delta \rho_{1},\;\Delta \rho_{2},\;\ldots ,\;\Delta \rho_{16},\;n_{x_{1}},\;n_{y_{1}},\;n_{z_{1}},d_{1},\ldots n_{x_{j}},\;n_{y_{j}},\;n_{z_{j}},d_{j}\right\}$		

**Table 4:** Estimated boresight angle error parameters.

Calibration Parameters	Scenario A	Scenario B	Scenario C
Δω (°)	0.0187 ± 0.0049	0.0285 ± 0.0082	–0.0012 ± 0.0061
Δφ (°)	–0.0155 ± 0.0037	0.0108 ± 0.0099	0.0507 ± 0.0205
Δκ (°)	0.0008 ± 0.0165	0.0135 ± 0.0038	0.0446 ± 0.0177

**Table 5:** RMSE values of different scenarios for the calibration procedure.

Method	Average of the Errors (%)
[11]	≈80
**Proposed method**	≈20
